# QSAR and Chemical Read-Across Analysis of 370 Potential MGMT Inactivators to Identify the Structural Features Influencing Inactivation Potency

**DOI:** 10.3390/pharmaceutics15082170

**Published:** 2023-08-21

**Authors:** Guohui Sun, Peiying Bai, Tengjiao Fan, Lijiao Zhao, Rugang Zhong, R. Stanley McElhinney, T. Brian H. McMurry, Dorothy J. Donnelly, Joan E. McCormick, Jane Kelly, Geoffrey P. Margison

**Affiliations:** 1 Beijing Key Laboratory of Environmental and Viral Oncology, Faculty of Environment and Life, Beijing University of Technology, Beijing 100124, China; baipy@emails.bjut.edu.cn (P.B.); fannie818@126.com (T.F.); zhaolijiao@bjut.edu.cn (L.Z.); lifesci@bjut.edu.cn (R.Z.); 2 Department of Medical Technology, Beijing Pharmaceutical University of Staff and Workers, Beijing 100079, China; 3 Chemistry Department, Trinity College, D02 PN40 Dublin, Ireland; tmcmurry@tcd.ie (T.B.H.M.); dor.donnelly@gmail.com (D.J.D.); 4 Carcinogenesis Department, Paterson Institute for Cancer Research, Manchester M20 9BX, UK; janekelly99@hotmail.com; 5 Epidemiology and Public Health Group, School of Health Sciences, University of Manchester, Stopford Building, Oxford Road, Manchester M13 9PG, UK

**Keywords:** MGMT, pseudosubstrates, inactivating agents, methyltransferase assay, MGMT activity determination, QSAR, read-across

## Abstract

*O*^6^-methylguanine-DNA methyltransferase (MGMT) constitutes an important cellular mechanism for repairing potentially cytotoxic DNA damage induced by guanine *O*^6^-alkylating agents and can render cells highly resistant to certain cancer chemotherapeutic drugs. A wide variety of potential MGMT inactivators have been designed and synthesized for the purpose of overcoming MGMT-mediated tumor resistance. We determined the inactivation potency of these compounds against human recombinant MGMT using [^3^H]-methylated-DNA-based MGMT inactivation assays and calculated the IC_50_ values. Using the results of 370 compounds, we performed quantitative structure–activity relationship (QSAR) modeling to identify the correlation between the chemical structure and MGMT-inactivating ability. Modeling was based on subdividing the sorted pIC_50_ values or on chemical structures or was random. A total of nine molecular descriptors were presented in the model equation, in which the mechanistic interpretation indicated that the status of nitrogen atoms, aliphatic primary amino groups, the presence of O-S at topological distance 3, the presence of Al-O-Ar/Ar-O-Ar/R..O..R/R-O-C=X, the ionization potential and hydrogen bond donors are the main factors responsible for inactivation ability. The final model was of high internal robustness, goodness of fit and prediction ability (*R*^2^_pr_ = 0.7474, *Q*^2^_Fn_ = 0.7375–0.7437, *CCC*_pr_ = 0.8530). After the best splitting model was decided, we established the full model based on the entire set of compounds using the same descriptor combination. We also used a similarity-based read-across technique to further improve the external predictive ability of the model (*R*^2^_pr_ = 0.7528, *Q*^2^_Fn_ = 0.7387–0.7449, *CCC*_pr_ = 0.8560). The prediction quality of 66 true external compounds was checked using the “Prediction Reliability Indicator” tool. In summary, we defined key structural features associated with MGMT inactivation, thus allowing for the design of MGMT inactivators that might improve clinical outcomes in cancer treatment.

## 1. Introduction

The DNA repair protein, *O*^6^-methylguanine-DNA methyltransferase (MGMT; also known as *O*^6^-alkylguanine-DNA alkyltransferase; AGT), can protect cells against the cytotoxic effects induced by DNA alkylating agents, such as the methylating antitumor drugs temozolomide (TMZ), procarbazine (PCB) and dacarbazine (DITC) and the chloroethylating antitumor drugs 1,3-bis(2-chloroethyl)-1-nitrosourea (BCNU), 1-(2-chloroethyl)-3-cyclohexyl-1-nitrosourea (CCNU) and 1-(4-amino-2-methyl-5-pyrimidinyl)methyl-3-(2-chloroethyl)-3-nitrosourea (ACNU) [1,2,3,4]. These alkylating agents predominantly exert their antitumor activity through the alkylation of DNA at the *O*^6^-position of guanine, generating a highly cytotoxic lesion [1,3,5]. However, *O*^6^-alkylguanine adducts can be repaired by MGMT that transfers an adducted alkyl group to its active center Cys145 residue in an irreversible and stoichiometric reaction [6]. Thus, MGMT is a “suicide enzyme” that acts only once, and further repair activity can be restored only by de novo protein synthesis [7].

**Figure 1:**
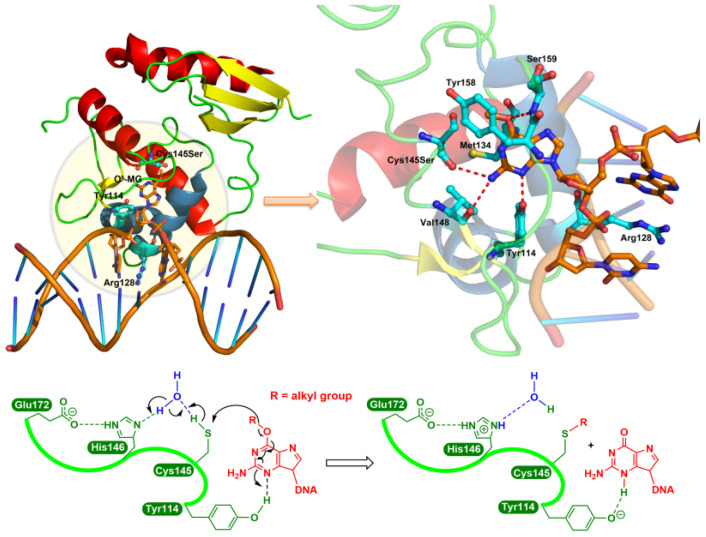
The consensus repair mechanism of *O*^6^-alkylguanines by MGMT. In the crystal structure of the Cys145Ser MGMT mutant bound to *O*^6^-MeG-containing DNA (upper left panel), the guanine moiety is flipped by Arg128 into the active site pocket and then forms hydrogen bonds (red dashed line; upper right panel) with Cys145Ser, Val148, Tyr114 and Ser159 residues. Lower panel: as a water-mediated general base, His146 deprotonates Cys145 (in the native protein), resulting in the transfer to the S atom of the *O*^6^-alkyl carbon, while the N3 is protonated by the Tyr114 residue.

The consensus repair mechanism by MGMT is shown in Figure 1. The formation of the *S*-alkyl adduct, at least in the case of methyl, causes a conformational change in MGMT, resulting in an increased recognition by ubiquitin ligase, targeting it for proteasome degradation [8,9,10].

Given the role of MGMT in alkylating chemotherapeutic resistance and its ability to act on *O*^6^-alkylguanines as free bases, various groups have synthesized a large number of such “pseudosubstrates” as potential inactivators of MGMT function [11,12,13,14,15,16,17,18,19,20,21,22]. Administering such agents prior to alkylating agents was proposed to ablate the protection provided by MGMT and hence increase the effectiveness of the chemotherapeutics [1,3,6].

Currently, only two MGMT inactivators, *O*^6^-benzylguanine (*O*^6^-BG) and *O*^6^-(4-bromothenyl)guanine (*O*^6^-4-BTG; Lomeguatrib), have been used as potentiating agents in clinical trials [6,23,24,25,26]. Unfortunately, the combination greatly increased the systemic toxicity of the alkylating chemotherapeutic drugs, requiring a considerable reduction in their dose [1,6,23,24,25]. This dose reduction might explain, at least in part, why the MGMT inactivators did not improve the clinical outcome of chemotherapy. Other factors might include: the rates of recovery of MGMT activity following depletion; tumor cell proliferation rates; the contribution of other protective mechanisms to cell survival; the relatively lower affinity of MGMT for free bases compared with *O*^6^-alkylguanines in duplex DNA [9,27]; poor water solubility; low bioavailability, instability and/or catabolic processes and rapid plasma clearance [28].

Given that the systemic delivery of MGMT inactivators exacerbates collateral toxicities, the synthesis of tumor-targeting inactivating agents might be expected to circumvent this [1,3,21,29,30]. To build into an inactivating agent tumor-targeting moieties, or, indeed, any other structural moieties that may optimize in vivo effectiveness, it will be essential to know what structural features endow the greatest activity and which regions cannot be modified without a loss of function.

To achieve this, we performed quantitative structure–activity relationship (QSAR) modeling to establish the detailed relationship between the molecular structure and MGMT inactivation potency. Although we reported a QSAR model for MGMT inhibitors in a previous study, the focus was primarily on base analogs, and the dataset used was relatively smaller, consisting of 134 compounds [16]. In the current study, we used MGMT in vitro inactivation assay results for a total of 370 compounds, which provided IC_50_ values as the response endpoint, which included not only base analogs but also other types of molecules. Additionally, all the experimental values for the 370 compounds were directly determined in our laboratory, rather than relying on data from the literature. Furthermore, the chemical synthesis of the 370 compounds was conducted within our lab as well. Our model contributes to a definitive mechanistic interpretation but also provides a tool for predicting and rapidly designing new candidates for depleting MGMT activity, including the tumor-targeting MGMT inactivators.

## 2. Materials and Methods

### 2.1. Compound Design and Synthesis

The listed compounds were designed by R. Stanley McElhinney and T. Brian H. McMurry and synthesized by members of the Chemistry Department of Trinity College, Dublin. Typical examples of the methods for the synthesis and analysis of the compounds are presented elsewhere [18]. In addition to curiosity-driven compounds in pursuit of increasingly potent agents, others were designed with specific objectives in mind, among which there were: combination alkylators–inactivators; tumor receptor targeting agents or potential precursors and agents that would produce antimetabolites upon dealkylation (see Table S1).

### 2.2. MGMT Activity Assay

Compounds were assayed for their ability to inactivate human recombinant MGMT in vitro using [^3^H]-methylated-DNA-based MGMT inactivation assays, as described in [18], at the Paterson Institute for Cancer Research, Manchester, U.K. The IC_50_ values were obtained for QSAR modeling. It should be noted that the in vitro assay does not differentiate between actual inactivation due to alkyl group transfer to Cys145 (see Figure 1) and competitive inhibition: alkyl group transfer to MGMT has been demonstrated for very few compounds, but we are not aware of any reports in which the mechanism of action has been proven to be competitive inhibition.

**Figure 2:**
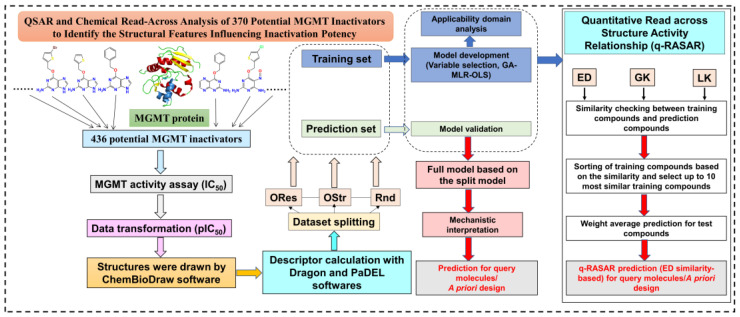
Workflow diagram for the methodology followed in the current study.

A flowchart of the methodology in the present study is shown in Figure 2.

### 2.3. Dataset Preparation

The Organization for Economic Co-operation and Development (OECD) principle 1 states that a QSAR model should be associated with “a defined endpoint” [31,32,33], and in the present study, the IC_50_ value was used as the activity endpoint. All IC_50_ values (μM) were transformed into –logIC_50_ (pIC_50_, mol/L), which is a common practice in QSAR modeling [32,34,35]; thus, the higher the pIC_50_ value, the more potent the MGMT inactivator.

### 2.4. Descriptor Calculation and Dataset Splitting

All the molecular structures were manually drawn using the ChemBioDraw Ultra 14.0 software (version 14.0, Cambridge soft, Cambridge, MA, USA) and geometrically optimized by energy minimization using its 3D module. After optimization, five quantum chemical descriptors including the dipole moment (*µ*), total energy (*E*), lowest unoccupied molecular orbital energy (*E*_LUMO_), highest occupied molecular orbital energy (*E*_HOMO_) and *E*_LUMO_ − *E*_HOMO_ gap were calculated. Dragon software (version 7.0) [36] and PaDEL-Descriptor software (version 2.18) [37] were used to calculate the molecular descriptors. In order to avoid the occurrence of conformational complexity due to the inclusion of 3D descriptors, and for the ease of interpretability and reproductivity, only 2D descriptors with a definite physicochemical meaning were calculated. To remove redundant variables, we excluded the constant or near-constant descriptors (>80% compounds have the same value) and inter-correlated descriptors (>0.95) from the descriptor pool.

To avoid possible bias, the dataset was split into training sets and prediction sets in an approximately 3:1 ratio using QSARINS v2.2.4 software (Varese, Italy) [38,39], in which the training set was used to establish the model, while the prediction set was used for model validation. Three splitting techniques were used [38], and in each, the inactivators with the maximum and minimum response pIC_50_ values and principal component 1 (PC1) scores were always put into the training set to cover the range of the prediction set. Splitting was undertaken by the software and was based on (1) the sorted pIC_50_ values (ORes) or (2) the structure based on the PC1 score of descriptors (OStr) or (3) was random. For ORes splitting, compounds were sorted by their pIC_50_ values, and from the second molecules, every fourth compound was placed in the prediction set, and the remaining three of the four were put into the training set. For OStr splitting, compounds were sorted by their PC1 scores, and again, one of every four compounds was placed in the prediction set. The distribution of splitting (PC1 vs. PC2) was checked by the principal component analysis (PCA) using only descriptor variables (Figure S1).

The dataset splitting methods ensured that the selection procedure was unbiased. In order to develop a model with a wider applicability domain (AD), once the best variable combination was found by the splitting technique, the full model was obtained through recalculation on the complete set (combining training and prediction sets), since all available experimental information was then considered [40,41].

### 2.5. Model Development and Validation

Variable selection from the large pool of descriptors is a very important step in the process of model development. Here, we used a Genetic Algorithm Variable Subset Selection (GA-VSS) tool of the QSARINS software [38] to conduct the variable selection. Initially, all the possible combinations of two descriptors were explored by all subset facilities to find the subset of descriptors encoding the response. Then, using the leave-one-out cross-validated correlation coefficient (*Q*^2^_LOO_) as a fitness function, GA-VSS was utilized to seek the new combinations with additional descriptors to yield the models. The generation per size, population size and mutation rate were given values of 2000, 200 and 20, respectively.

Depending on the empirical ratio [33,42], to reduce the possibility of chance correlation, the number of descriptors in the model should be less than one-fifth of the number of training compounds. QSAR models were established through Multiple Linear Regression (MLR) using the Ordinary Least Squares (OLS) approach implemented in the QSARINS software [38]. According to the OECD principle 2, a QSAR model should be associated with “an unambiguous algorithm” [42], which ensures the transparency of the model algorithm. It should be noted that the algorithmic information in commercial models is usually less publicly available.

Depending on the OECD principle 4, a QSAR model should be associated with “appropriate measures of goodness-of-fit, robustness and predictivity” [42]. The internal robustness and predictive ability of the model were assessed by the *Q*^2^_LOO_, *Q*^2^_LMO_, *R*^2^ (including adjusted *R*^2^_adj_), root mean standard error (*RMSE*_tr_) and mean absolute error (*MAE*_tr)_ [43,44]. In the leave more out (LMO) procedure, 30% of compounds were excluded from each calculation for 2000 iterations. A Y-randomization test (the dependent variable Y was randomly scrambled, while the independent variable matrix is unchanged) with 2000 iterations was also used for assessing the chance correlation between the model descriptors and response endpoint. In this test, the sequence of the response vector Y was randomly scrambled, while the descriptor variable X for each object was unchanged. In addition, we set the threshold of the QUIK (Q Under Influence of K) rule as 0.05 to exclude multi-co-linearity [42,45]. The external predictivity of the model was evaluated by the statistical parameters *R*^2^_pr_, *Q*^2^_F1_, *Q*^2^_F2_, *Q*^2^_F3_, *CCC*_pr_, *RMSE*_pr_ and *MAE*_pr_ [44]. The detailed calculated formulae can be found elsewhere [44,46]: all the parameters are listed in Table S2 in the Supplementary Materials.

### 2.6. Best Model Selection by Multiple-Criteria Decision Making

On the basis of fitting and internal and external validation, the Multiple-Criteria Decision Making (MCDM) module implemented in QSARINS software [38] was utilized to rank the model performance as a score from 0 (the worst) to 1 (the best). The MCDM_fit_ value was computed via the maximization of *R*^2^, *R*^2^_adj_ and *CCC*_tr_, whereas the minimization of the *R*^2^ − *R*^2^_adj_. MCDM_ext_ value was computed via the maximization of *Q*^2^_F1_, *Q*^2^_F2_, *Q*^2^_F3_ and *CCC*_pr_. As a consequence, we selected the best QSAR model depending on both the MCDM_fit_ and MCDM_ext_ values. These models fulfill the OECD principles as well as various validation criteria [42]. It is accepted that the best model should be obtained with the lowest number of descriptors.

### 2.7. Applicability Domain (AD) Analysis

Depending on the OECD principle 3, a QSAR model should have “a defined domain of applicability” [42]. Only the compounds inside the AD of the model should provide reliable predictions. Here, we used both leverage and standardized residue approaches to define the AD [38,39]. Structural outliers were identified using the leverage approach. If a compound has a hat (*h*) value greater than the warning h*, it will be identified as a structural outlier. The warning h* value was calculated by the formula of 3(*p* + 1)/*n*, in which *p* is the number of variables in the model equation, and *n* is the number of training set compounds. If the standardized residual of a compound is more than three standard deviation units, it is identified as a response outlier.

We also prepared a true external set consisting of 66 compounds for checking the predictivity of the developed model. In order to visually show the prediction confidence for each molecule, an Insubria graph which plots the predicted values of the training/true external set against their hat values was generated [39]. The predictions for compounds with hat values greater than h* should be considered to have low confidence.

### 2.8. Prediction Using a Similarity-Based Chemical Read-Across Technique

Read-Across (RA) is a completely similarity-based technique without the process of developing a statistical model, which is the most significant feature that is different from the classical QSAR methodology [47,48]. RA is widely used in qualitive predictions; however, the quantitative read-across technique was also reported in recent years. To further improve the external predictive ability, we used a novel approach called the quantitative read-across structure–activity relationship (q-RASAR) [49,50]. After completing the development of 2D-QSAR, the training set was divided into a subtraining set and subtest set, followed by the optimization of the hyperparameter using the Read-Across V4.1 tool (https://sites.google.com/jada vpuruniversity.in/dtc-lab-software/home) (accessed on 1 April 2023). The optimized hyperparameters were applied to the original dataset files as the input. In this study, the similarity determination between the training compounds and test compounds was determined based on the Euclidean distance, Laplacian kernel function and Gaussian kernel function. Then, we calculated the RASAR descriptors based on the selected descriptors in the 2D-QSAR model by the RASAR-Desc-Calc-v2.0 software (https://sites.google.com/jada vpuruniversity.in/dtc-lab-software/home) (accessed on 1 April 2023) using the optimized hyperparameters. The RASAR descriptors were combined with original 2D descriptors to develop the q-RASAR model using the same setting as 2D-QSAR. Finally, we obtained a q-RASAR model and q-RASAR-full model; the latter was also applied to the predictions of true external compounds.

## 3. Results and Discussion

### 3.1. MGMT Inactivation

The MGMT inactivation assay results, along with the compound name, number and structure, are listed in Table S1 in the Supplementary Materials.

### 3.2. Chemical Space Distribution

After processing the original data, we obtained 458 entries for MGMT inactivation (Table S1). For compounds also produced as salts, only the entry corresponding to the free compound was used. For hydrates, we deleted the water molecules in the descriptor calculations and model development.

The initial QSAR model development showed that 17 compounds were always response outliers that substantially influenced the linear fitting in different dataset splitting schemes. These compounds may be related to the activity cliffs [51,52]. It is suspected that the experimental IC_50_ values for these compounds may be erroneous, and they were therefore excluded. In addition, there were 49 compounds that were found to not inactivate MGMT at the highest concentration used in the assay and thus had no definitive IC_50_ values. Therefore, a total of 66 compounds were excluded from the training and prediction sets and selected as the true external set. Hence, in our modeling study (Table S1), the numbers for the training set, prediction set and true external set were 279, 91 and 66, respectively, in the best QSAR model (Tables S3 and S4).

**Figure 3:**
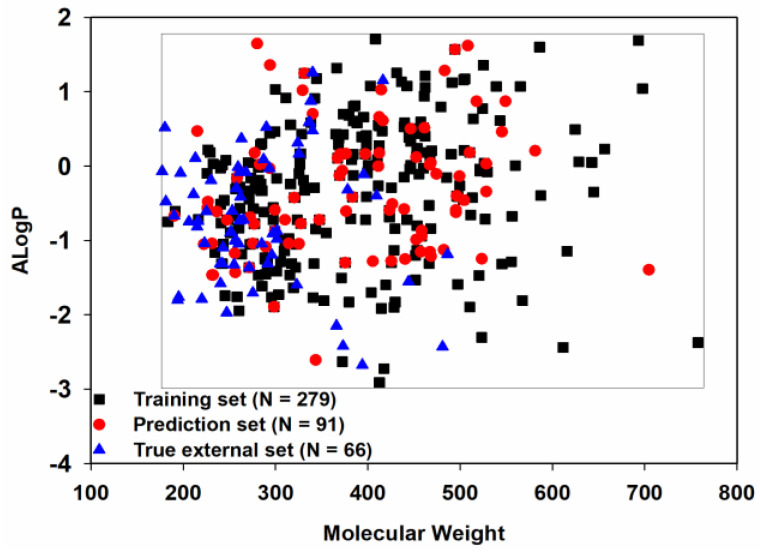
Chemical space distribution of the three datasets.

Chemical space similarity is very important for evaluating the predictive performance of a model. Here, we used two commonly used physicochemical parameters: molecular weight (MW) and Ghose−Crippen LogK_ow_ (ALogP), to explore the chemical space distribution [53,54,55,56] and plotted these as a scatter diagram (Figure 3).

Given that the training set, prediction set and true external set, as expected, shared a similar chemical space, the models derived from the training set should have a broad applicability domain (AD) and thus a good degree of generalization.

### 3.3. QSAR Modeling of Potential MGMT Inactivators

#### 3.3.1. Model Selection and Evaluation

According to the criteria recognized by Golbraikh and Tropsha [43], if a QSAR model meets the following thresholds for different statistical parameters: *Q*^2^_LOO_ > 0.5, *R*^2^ and *R*^2^_pr_ > 0.6; 0.85 ≤ *k* or *k*′ ≤ 1.15; |*R*^2^_0_ − *R*′^2^_0_| < 0.3, it should be considered an acceptable model. *R*^2^_0_ and *R*′^2^_0_ represent the correlation coefficients of regression of the predicted versus experimental values and experimental versus predicted values through the origin, respectively. *K* and *k*′ represent the slopes of the corresponding regression lines for *R*^2^_0_ and *R*′^2^_0_, respectively.

Of the three splitting methods, that based on the ORes model had low values of *Q*^2^_LOO_ and R^2^ and did not meet the basic standard for an acceptable model [43]. This may be due to the model (Equation (1), Table 1) containing only three descriptors, and this cannot adequately simulate the biochemical response endpoint (pIC_50_).

Although the OStr model (Equation (2), Table 1) is internally robust and stable (*Q*^2^_LOO_ = 0.6496, *R*^2^ = 0.6826), its external predictive performance is compromised (*R*^2^_pr_ < 0.6) according to statistical criteria [43]. On the other hand, the OStr model included 13 molecular descriptors, which complicates the interpretation of the model.

**Figure 4:**
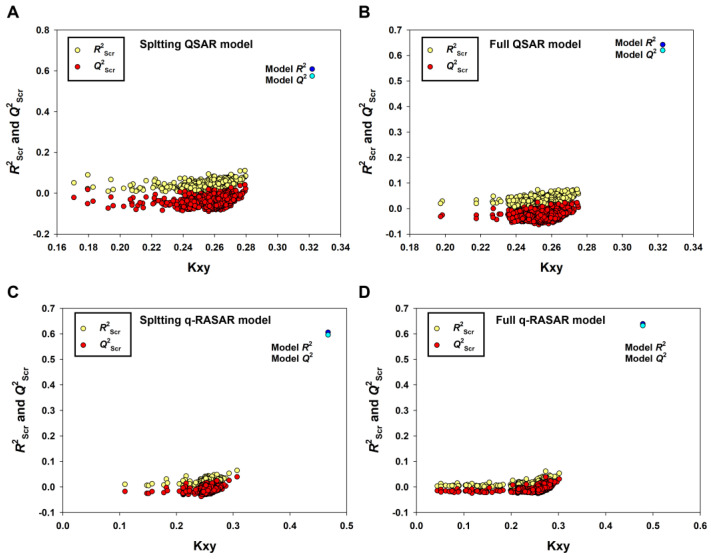
The results of Y-randomization for the Random splitting QSAR model (**A**), the Full QSAR model (**B**), the Random splitting q-RASAR model (**C**) and the Full q-RASAR model (**D**).

It is remarkable that only the model derived from the Random splitting method (Rnd model or 2D-QSAR) fulfilled the Golbraikh and Tropsha criteria [43]. Furthermore, the 2D-QSAR model (Equation (3), Table 1) had the best predictivity for the prediction set (*R*^2^_pr_ = 0.7474, *Q*^2^_Fn_ = 0.7375~0.7437, *CCC*_pr_ = 0.8530), which met even the higher statistical standard proposed by Chirico and Gramatica [44], in which the thresholds of *Q*^2^_Fn_, *R*^2^_pr_ and *CCC*_pr_ are 0.7, 0.7 and 0.85, respectively. Low values of *Q*^2^_Yscr_ (−0.0424) and *R*^2^_Yscr_ (0.0324) indicated that the model was not generated by chance correlation (Figure 4A).

In fact, we also tried to use other dataset splitting methods such as Kennard–Stone (http://teqip.jdvu.ac.in/QSAR_Tools/) (accessed on 16 August 2023) and other modeling methods (PLS or stepwise MLR implemented in the Double Cross-Validation v2.0 Software Tool) [57] for model development. However, the quality of these models was not better than that of the Ran (2D-QSAR) model.

**Figure 5:**
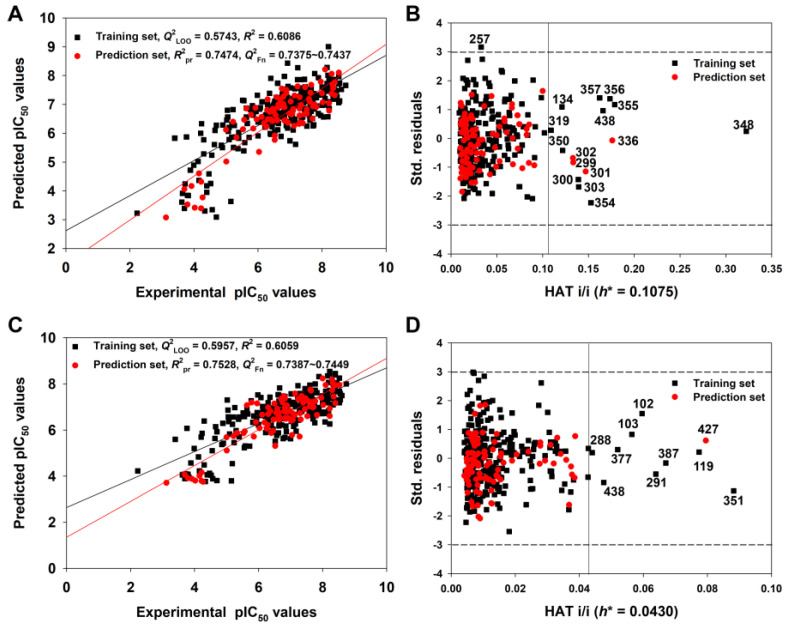
The graph of experimental versus predicted pIC_50_ values (**A**) and the Williams plot (**B**) for the 2D-QSAR model defined by Equation (3); the graph of experimental versus predicted pIC_50_ values (**C**) and the Williams plot (**D**) for the q-RASAR model defined by Equation (5) (Table 1).

Figure 5 shows the graph of experimental versus predicted pIC_50_ values (Figure 5A) and the Williams Plot (Figure 5B) for the AD analysis of the 2D-QSAR model derived from the Random splitting method.

We found that the training and prediction set compounds were homogenously distributed around the trend line, indicating a good predictive ability for query molecules. Considering the AD of the 2D-QSAR model (Figure 5B), only four compounds in the prediction set and eleven compounds in the training set had hat values greater than the warning h* value (0.108). These were identified as structural outliers, and they may thus be influential in the variable selection in the training set. However, we are not suggesting that these structural outliers cannot be predicted reliably. For example, compound **348** has the maximum hat value, but its predicted residual was very small (0.1601 log unit) (see the detailed data in Table S3). The four prediction set compounds (**299**, **301**, **302**, **336**) were also predicted accurately since their predicted residuals were also small (Table S3). Meanwhile, the predicted residuals of compounds **355**, **356** and **357** were relatively higher (~1 log unit) (Table S3). In contrast, only one compound, **257**, was identified as a response outlier because it had standardized residuals greater than 3.0 standard deviation units (Table S3).

Table 2 described the nine molecular descriptors selected by the GA-VSS that were present in the model equation along with their relative importance (Std. coefficient) and physicochemical definitions.

#### 3.3.2. Full Model

As described above, we have verified the external predictive ability of the 2D-QSAR model with the best combination of descriptor variables. Subsequently, based on the same variables, the model (Equation (3), Table 1) was recalibrated using the entire set of compounds (N_tr_ = 370). The new model was called the 2D-QSAR-Full model (Equation (4), Table 1) and it considered all the available information in the training and test sets.

**Figure 6:**
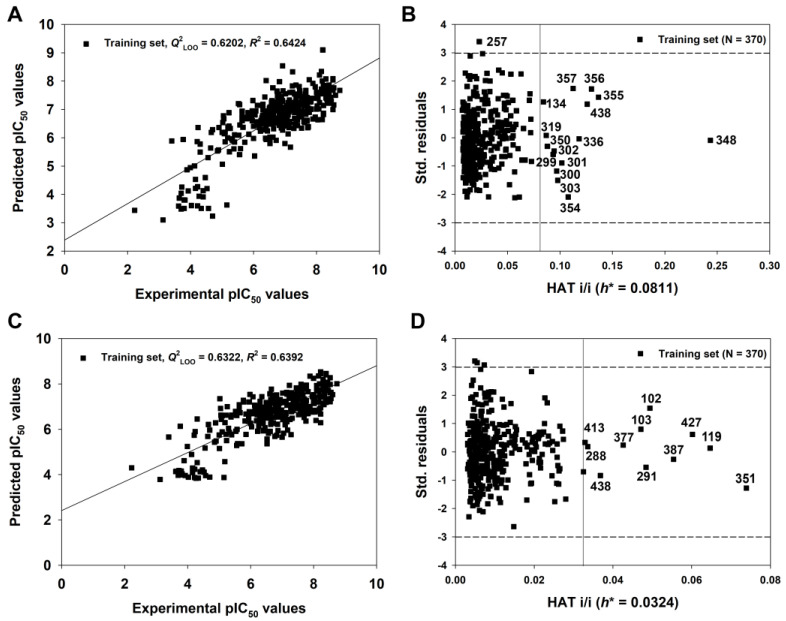
The graph of experimental versus predicted pIC_50_ values (**A**) and the Williams plot (**B**) for the 2D-QSAR-Full model defined by Equation (4) in Table 1; the graph of experimental versus predicted pIC_50_ values (**C**) and the Williams plot (**D**) for the q-RASAR-Full model defined by Equation (6) in Table 1.

The endpoint, expressed as pIC_50_ (−logIC_50_, mol/L), ranged from 2.22 to 8.74, spanning more than six log units and suggesting that the dataset is adequate for QSAR studies. As shown in Table 1, the 2D-QSAR-Full model showed satisfactory internal fitness (*R*^2^ = 0.6426) and robustness (*Q*^2^_LOO_ = 0.6202, *Q*^2^_LMO_ = 0.6127). Again, the values of *Q*^2^_Yscr_ (−0.0309) and *R*^2^_Yscr_ (0.0248) were very low, indicating the absence of any chance correlation. The graph of experimental versus predicted pIC_50_ values (Figure 6A) and the Williams Plot (Figure 6B) are given below.

**Figure 7:**
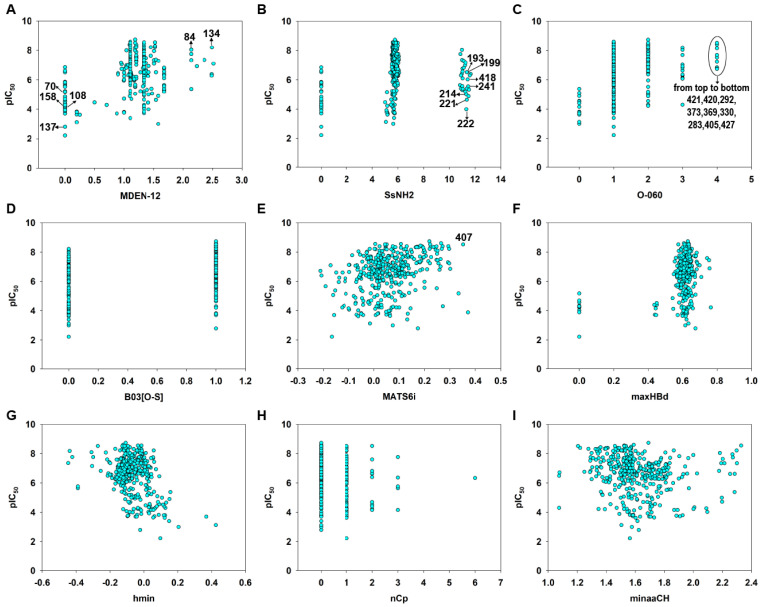
Variable scatter plot of each descriptor versus pIC_50_. MDEN-12 (**A**), SsNH2 (**B**), O-040 (**C**), B03[O-S] (**D**), MATS6i (**E**), maxHBd (**F**), hmin (**G**), nCp (**H**) and minaaCH (**I**).

Molecular descriptors were calculated from the 2D structural information using Dragon [36] and PaDEL [37] software. We emphasize that these descriptors capture the global properties of the molecular structure or encode for some specific groups or fragments, such as electronic accessibility (E-State), spatial autocorrelations (2D autocorrelation) or the presence or absence of a specific fragment. The scatter plot of each descriptor versus pIC_50_ was shown in Figure 7. The value of each descriptor was listed in Table S5 in the Supplementary Materials.

According to the model Equation (4) (Table 1) and the standardized coefficients of each variable (Table 2), the most important descriptors for MGMT inactivation were MDEN-12 (std. coefficient 0.6567) and SsNH2 (std. coefficient −0.4582). It should be noted that MDEN-12 was positively correlated (Figure 7A), while SsNH_2_ was negatively correlated with the MGMT inactivation potency (Figure 7B). MDEN-12 is the molecular distance edge between all primary and secondary nitrogens [58]; for example, compounds **84** and **134** with high MDEN-12 values (2.144 and 2.490, respectively) were strong inactivators (pIC_50_ = 8.04 and 8.20, respectively). Figure S2 showed MDEN-12 descriptor values for the two benchmark inactivators Lomeguatrib and O^6^-BG and the selected compounds. This descriptor also highlights the importance of the presence of –NH_2_: compounds without 2′-NH_2_ (such as **70**, **108**, **111** and **158**) were commonly less effective in MGMT inactivation. This is consistent with our previous study indicating that the 2′-NH_2_ of guanine is essential for inactivation because it plays an important role in hydrogen bond formation with Cys145/Val148 residues of MGMT (see Figure 1) [3,8,59]. However, MDEN-12 as a single descriptor did not adequately model the MGMT inactivation potency in a general model; hence, the GA-VSS selected additional descriptors to obtain a model with higher predictivity. SsNH2 represents the sum of atom-type electrotopological states (E-State): –NH_2_ [58], indicating that the aliphatic primary amino can compromise the MGMT inactivation potency to a certain extent, especially for the guanine derivatives **193**, **199**, **214**, **221**, **222**, **241** and **418,** which have an aliphatic amino in the N9 position. This was also supported by a previous study indicating that a large polar group at the N9 position of guanine was not well tolerated [14]. Figure S3 showed SsNH2 descriptor values for Lomeguatrib, O^6^-BG and the selected compounds. Indeed, MDEN-12 and SsNH2 were two mutually balanced descriptors in the model, as indicated by the compounds **64**, **65** and **66**, since they had low values (0) for the two opposite descriptors, but a moderate potency (pIC_50_ = 5.72, 6.52 and 4.55, respectively) (see Table S5).

O-060 (std. coefficient 0.2012) and B03[O-S] (std. coefficient 0.2452) are two descriptors that are related to the presence of a specific group or fragment [58], and on the basis of these coefficients, they were positive contributors to MGMT inactivation potency. O-060 belongs to the basic descriptors of atom-centered fragments, representing the presence of Al-O-Ar/Ar-O-Ar/R..O..R/R-O-C=X fragments. In the entire dataset, this descriptor had discrete values of 0, 1, 2, 3 and 4, respectively. There were nine compounds (**283, 292, 330, 369, 373, 405, 420, 421** and **427**) that had the maximum O-060 value of four and a relatively high MGMT inactivation activity (pIC_50_ = 6.764–8.520) (Figure 7C). The values of the O-060 descriptor for Lomeguatrib, *O*^6^-BG, and the selected compounds are shown in Figure S4. B03[O-S] indicates the presence or absence of O-S at topological distance 3, and it was clear that the thiophene group substituted on the guanine O^6^ position in compounds like **112**, **401** and **402** (Tables S1 and S5) contributed substantially to their high inactivation potency (Figure 7D). Figure S5 shows B03[O-S] values for Lomeguatrib, *O*^6^-BG and the selected compounds.

MATS6i (std. coefficient 0.1946) is the Moran autocorrelation of lag 6 weighted by the ionization potential [58]. It indicates the relative charge distribution of a molecule, i.e., the electron cloud, and thus may enhance charge or hydrogen bond interactions with the target. Because base analog-mediated MGMT inactivation is absolutely dependent on the ability to donate a carbocation to the active site of MGMT [1,3,6], MATS6i is generally a positive contributor to the response endpoint (Figure 7E). Figure S6 showed MATS6i values for Lomeguatrib, *O*^6^-BG and the selected compound **407**, which had a high MATS6i value (0.352) and a high inactivation potency (pIC_50_ = 8.523) (Table S5).

The descriptor maxHBd (std. coefficient 0.1850) indicates the maximum E-States for (strong) hydrogen bond donors and clearly contributes to increasing the inactivation activity (Figure 7F). For example, compounds **411** and **90** were strong inactivators (pIC_50_ = 8.55 and 8.52, respectively) with high maxHBd values (0.629 and 0.637, respectively) (see Table S5). Figure S7 shows the values of the maxHBd descriptor for Lomeguatrib, *O*^6^-BG and the selected compounds.

The last three descriptors were nCp (−0.1331), hmin (std. coefficient −0.1792) and minaaCH (−0.1063) (see Table 2), the latter two being E-state descriptors [58]. Individually, these three descriptors were less important in defining the model equation but supported the six main descriptors. The descriptor hmin indicates a minimum H E-State, which encodes for the minimum E-State of hydrogen atoms. The minaaCH descriptor represents the minimum atom-type E-State aromatic-CH-aromatic, in which atom-type E-state indices are computed by summing the E-state values of all atoms of the same atom type in a molecule [58]. These descriptors characterize the information related to the electronic accessibility of an atom and hence the probability of intermolecular interactions [60]. In our model, the two E-state descriptors were negatively correlated with the response endpoint, which was consistent with the aquatic toxicity models of pesticide and pharmaceuticals [41,61]. The nCp descriptor represents the number of terminal primary sp3 carbons [58], and this was also inversely related to the response according to its equation coefficient. The values of the three descriptors for Lomeguatrib, *O*^6^-BG and the selected compounds are shown in Figure S8.

The developed model was derived from multivariable combinations based on a statistically driven procedure (i.e., GA-VSS-based selection). Thus, none of the descriptors can independently explain the distribution of the modeled endpoint, and only the combination of all selected descriptors can accurately model the response to be studied.

### 3.4. q-RASAR Analysis

After the development of the 2D-QSAR model, the same training and test set files were used as inputs for quantitative Read-Across predictions using three different similarity-based functions, namely, the Euclidean Distance, Gaussian Kernel function and Laplacean Kernel function [47,48]. For the predictions of prediction set compounds, a default sigma value (σ) of 1 for the Gaussian kernel function and a default gamma value (γ) of 1 for the Laplacian kernel function were used, and the distance threshold value and similarity threshold value were set as 1 and 0, respectively. The number of the closest training compounds for activity prediction was six. It was found that the external validation parameters like *Q*^2^_F1_ (0.7401), *Q*^2^_F2_ (0.7399), *RMSE*_pr_ (0.6187) and *MAE*_pr_ (0.4802) from quantitative Read-Across using the Euclidean Distance (see Table S6) were better than those of 2D-QSAR.

To establish QSAR-based Read-Across predictions, we performed the q-RASAR modeling [49,50]. The equation of the q-RASAR model (Equation (5)) is listed in Table 1. In Equation (5), the RA function (ED) variable was a Euclidean Distance-based Read-Across prediction function obtained from the original 2D descriptors. It can be accessed by the free online tool RASAR-Desc-Calc-v2.0 (https://sites.google.com/jadavpuruniversity.in/dtc-lab-software/home) (accessed on 1 April 2023). The low difference between *R*^2^ and *Q*^2^_LOO_ indicated the robustness of the model and the higher values of *R*^2^_pr_, *Q*^2^_F1_, *Q*^2^_F2_ and *Q*^2^_F3_, and the lower *MAE*_pr_ value suggested the good predictivity and transferability of the q-RASAR model. Due to the good internal robustness and external predictivity, we also constructed the q-RASAR-full model (Equation (6) in Table 1) using all the available information. Low values of *Q*^2^_Yscr_ and *R*^2^_Yscr_ (0.0324) indicated that the q-RASAR model and q-RASAR-Full model were not generated by chance correlation (Figure 4C,D).

The linear correlations for the q-RASAR and q-RASAR-Full models are shown in Figure 5C and Figure 6C, respectively. Meanwhile, the AD analysis of the q-RASAR and q-RASAR-Full models is shown in Figure 5D and Figure 6D. We found relatively fewer outliers in q-RASAR modeling compared to 2D-QSAR modeling.

The detailed information about the q-RASAR model is listed in Table S7 in the Supplementary Materials. The values of each variable in the q-RASAR-Full model are listed in Table S8 in the Supplementary Materials.

### 3.5. Application of the 2D-QSAR-Based Full Model and q-RASAR-Full Model

**Figure 8:**
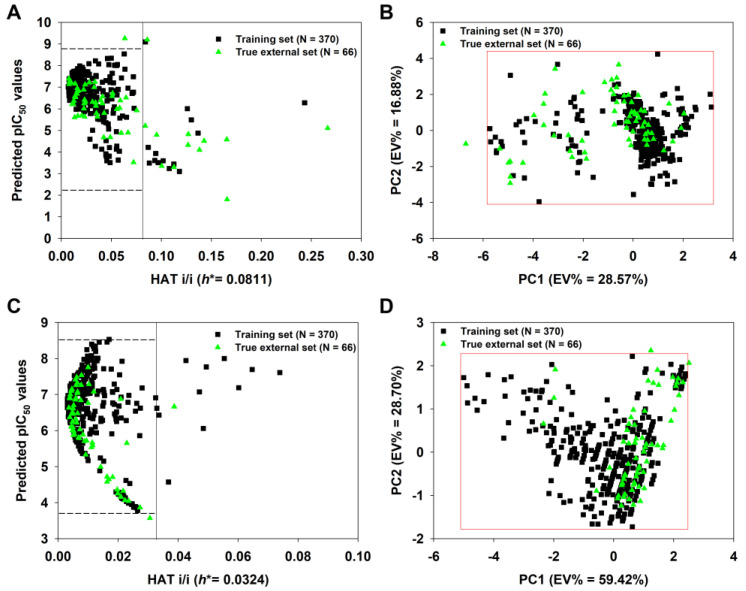
Insubria graph of the 2D-QSAR-Full model (**A**) and principal component analysis (PCA) plot based on the selected nine descriptors shown in Table 2 (**B**); Insubria graph of the q-RASAR-Full model (**C**) and PCA plot based on the three variables shown in Equation (6) (**D**). The four plots show the applicability domain (AD) when the two Full models are applied to true external compounds without experimental values.

We constructed a true external set consisting of 66 unknown molecules. After calculating their descriptors, the 2D-QSAR-Full model was applied to predict their pIC_50_ values. As shown in Figure 8A, 12 of 66 true external compounds lay outside the model’s AD, since their *h* values are greater than h* (0.081), suggesting >80% prediction coverage. In particular, compound **36** has the highest *h* value (0.2664 >> h*). If we defined the AD of the model using the PCA approach (Figure 8B), only one compound (again, compound **36**) in the true external set falls outside the AD, resulting in a more significant prediction coverage (98.5%).

Similarly, the q-RASAR-Full model was also applied to the true external compounds. As shown in Figure 8C, only one true external compound (**436**) lay outside the model’s AD, suggesting >98% prediction coverage. In fact, compound **436** only has a slightly higher *h* value (0.0387) compared to the threshold value h* (0.0324). Using the PCA approach (Figure 8D), only one compound (compound **308**) in the true external set falls outside the AD, also showing a considerable prediction coverage.

Subsequently, we also used the “Prediction Reliability Indicator” tool (http://dtclab.webs.com/software-tools) (accessed on 22 December 2022) [62] to check the prediction quality for each true external compound. Each compound is scored (composite score of 3, 2 or 1) based on the absolute prediction errors that correspond to “Good”, “Moderate” or “Bad or Unreliable” prediction quality, respectively. As shown in Table S9, we found 56 “Good” compounds, 10 “Moderate” compounds and no “Bad or Unreliable” compounds derived from the 2D-QSAR-Full model. As for the q-RASAR-Full model, we found 61 “Good” compounds, 5 “Moderate” compounds and no “Bad or Unreliable” compounds (Table S10). The results suggest that our Full models, especially the latter, have a wide and reliable prediction scope and that they can be used to forecast the MGMT inactivation potency of untested compounds. A priori designed compounds would be identified by our validated model and the most potent prioritized so that, time, money and resources would be saved. Of course, the multi-objective optimization modeling is also very important, especially when simultaneously considering the bioactivity, bioavailability and toxicity [63,64].

## 4. Conclusions

In this study, using the experimental IC_50_ values for a total of 370 MGMT inactivators, we developed QSAR models using a GA-MLR method, and Dragon and PaDEL software were combined to calculate molecular descriptors for model establishment. Three splitting models were assessed for robustness, reliability, fitness and predictivity. After selecting the best splitting model, a 2D-QSAR-Full model was then recalibrated using all the available experimental information (370 compounds). The mechanistic interpretation indicated that the status of nitrogen atoms, aliphatic primary amino groups, the presence of O-S at topological distance 3, the presence of Al-O-Ar/Ar-O-Ar/R..O..R/R-O-C=X, the ionization potential and hydrogen bond donors are the main factors controlling MGMT inactivation potency. Using the selected features in the 2D-QSAR and chemical Read-Across technique, we developed the q-RASAR model, which exhibited better external predictive ability. The AD analysis showed that the splitting 2D-QSAR model and 2D-QSAR-Full model had a significantly high coverage for the test set and true external set. In summary, the QSAR Full model developed in this study can be used for optimizing the design of novel MGMT inactivators. Thus, for novel untested compounds, we can predict their IC_50_ if they are located at the applicability domain, focus on compounds with a high inactivation potential and, hence, reduce unnecessary chemical synthesis.

## Figures and Tables

**Table 1:** Statistical parameters for the internal and external validation of the developed QSAR models ^+^.

Division			Fitting	Robustness		Chance Correlation		External Validation					Accuracy			
Scheme	N_tr_	N_pr_	*R* ^2^	*Q* ^2^_LOO_	*Q* ^2^_LMO_	*Q* ^2^_Yscr_	*R* ^2^_Yscr_	*R* ^2^_pr_	*Q* ^2^_F1_	*Q* ^2^_F2_	*Q* ^2^_F3_	*CCC*_pr_	*RMSE*_tr_	RMSE_pr_	*MAE*_tr_	*MAE*_pr_
ORes	278	92	0.5098	0.4968	0.4952	−0.0186	0.0108	0.5319	0.5271	0.5266	0.5658	0.6891	0.8669	0.8151	0.6666	0.6440
pIC_50_ = 3.9243 + 0.6729F09[O-S] + 0.121SaaN + 1.005MDEN-12 (*k* = 1.0, |*R*^2^_0_ − *R*′^2^_0_| = 0.4582)																(1)
OStr	278	92	0.6826	0.6496	0.6451	−0.0544	0.0432	0.5882	0.5721	0.5712	0.5814	0.7617	0.6917	0.7943	0.5514	0.5982
pIC_50_ = 7.1639 − 47.2151VE2sign_B(m) + 2.8662MATS6i − 1.5036GATS7p − 0.1826H-048 + 0.5367O-060 − 0.3698B08[N-O] + 0.3948F06[C-S] − 0.1856SsNH2 + 0.0537minHBint6 + 1.8451MDEN-12 + 0.1755MDEN-22 − 0.8906minaaCH (*k* = 1.0, |*R*^2^_0_ − *R*′^2^_0_| = 0.1428)																(2)
**Random (2D-QSAR)**	**279**	**91**	**0.6086**	**0.5743**	**0.5648**	**−0.0424**	**0.0324**	**0.7474**	**0.7377**	**0.7375**	**0.7437**	**0.8530**	**0.7682**	**0.6215**	**0.6114**	**0.5224**
**pIC_50_ = 4.5562 + 2.5829MATS6i − 0.191nCp + 0.3196O-060 + 0.6746B03[O-S]** **− 0.2499SsNH2 + 2.4853maxHBd − 2.3712hmin + 1.1784MDEN-12 − 0.6509minaaCH** (*k* = 1.0, |*R*^2^_0_ − *R*′^2^_0_| = 0.2436)																**(3)**
**2D-QSAR-Full model**	**370**	**—**	**0.6426**	**0.6202**	**0.6127**	**−0.0309**	**0.0248**	**—**	**—**	**—**	**—**	**—**	**0.7320**	**—**	**0.5855**	**—**
**pIC_50_ = 4.7334 + 2.3826MATS6i − 0.2387nCp + 0.3401O-060 + 0.6301B03[O-S] − 0.248SsNH2 + 2.1364maxHBd** **− 2.3442hmin + 1.8332MDEN-12 − 0.6324minaaCH**																**(4)**
**q-RASAR**	**279**	**91**	**0.6059**	**0.5957**	**0.5926**	**−0.0189**	**0.0103**	**0.7528**	**0.7389**	**0.7387**	**0.7449**	**0.8560**	**0.7708**	**0.6201**	**0.6144**	**0.4812**
**pIC_50_ = −1.1683 + 0.9192RA function (ED) + 0.0718CATS2D_07_AL + 1.2875LLS_02**																**(5)**
**q-RASAR-Full model**	**370**		**0.6392**	**0.6322**	**0.6305**	**−0.0136**	**0.0083**	**—**	**—**	**—**	**—**	**—**	**0.7354**	**—**	**0.5799**	**—**
**pIC_50_ = −1.1089 + 0.9149RA function (ED) + 0.0667CATS2D_07_AL + 1.3166LLS_02**																**(6)**

^+^ All abbreviations are explained in the text. The bold typefaces indicate the best splitting model and the recalibrated full model using the same descriptors. The q-RASAR model was also established based on the best splitting model and recalibrated as the q-RASAR-Full model.

**Table 2:** Descriptors selected by GA-VSS with the standardized coefficient, range of values and physicochemical definitions.

Descriptors	Std. Coefficient	Range		Definition
	(Full Model)	Min	Max	
MATS6i	0.2152 (0.1946)	−0.212	0.371	Moran autocorrelation of lag 6 weighted by ionization potential (DRAGON)
nCp	−0.106 (−0.1331)	0	6	number of terminal primary C(sp3) (DRAGON)
O-060	0.1908 (0.2012)	0	4	Al-O-Ar/Ar-O-Ar/R..O..R/R-O-C=X (Atom-centered fragments, Basic descriptors) (DRAGON)
B03[O-S]	0.2623 (0.2452)	0	1	Presence/absence of O-S at topological distance 3 (DRAGON)
SsNH2	−0.4636 (−0.4582)	0	11.662	Sum of atom-type E-State: –NH_2_ (DRAGON)
maxHBd	0.2085 (0.185)	0	0.764	Maximum E-States for (strong) Hydrogen Bond donors (PaDEL)
hmin	−0.1766 (−0.1792)	−0.447	0.425	Minimum H E-State (PaDEL)
MDEN-12	0.6452 (0.6567)	0	2.515	Molecular distance edge between all primary and secondary nitrogens (PaDEL)
minaaCH	−0.1103 (−0.1063)	1.075	2.329	Minimum atom-type E-State: CH: (PaDEL)

## Data Availability

Data will be available on request.

## Supplementary Materials

The following supporting information can be downloaded at: https://www.mdpi.com/article/10.3390/pharmaceutics15082170/s1. Figures S1–S8 and Tables S1–S10.
