# Supplementary material for: QSAR and Chemical Read-Across Analysis of 370 Potential MGMT Inactivators to Identify the Structural Features Influencing Inactivation Potency
Source: Pharmaceutics. 2023 Aug 21;15(8):2170. doi: 10.3390/pharmaceutics15082170 (PMC10458236; doi:10.3390/pharmaceutics15082170)
Supplement: Supplementary file 1 [file pharmaceutics-15-02170-s001.zip › Supplementary Materials-Figures S1-S8.pdf]

Supplementary Materials (Figures S1-S8)

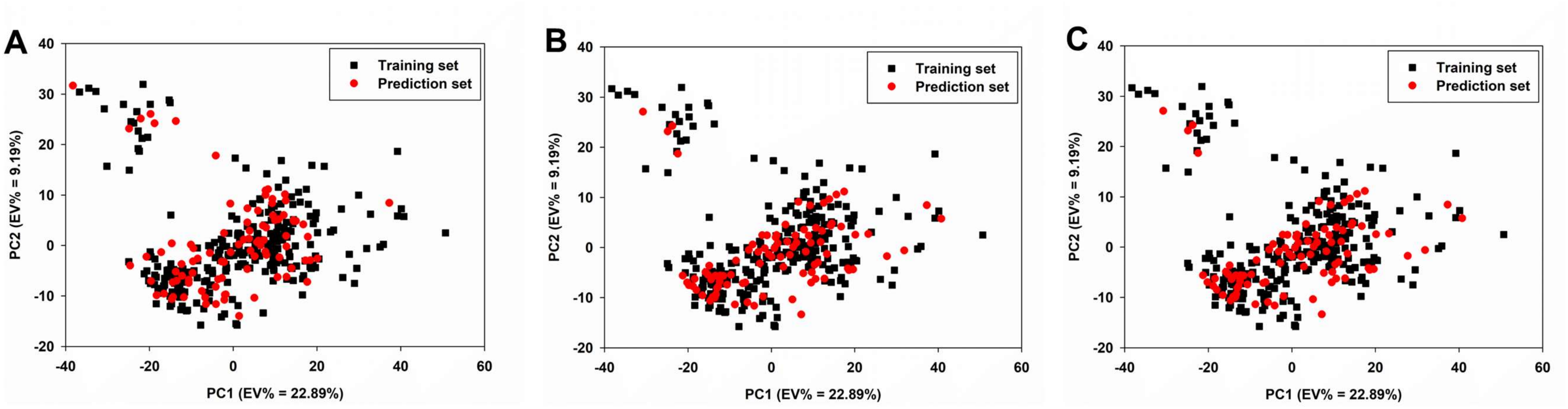

Figure S1. Principle component analysis plots for different dataset splitting methods. (**A**) ORes; (**B**) OStr; and (**C**) Random. Black squares: training set; Red circles: prediction set.

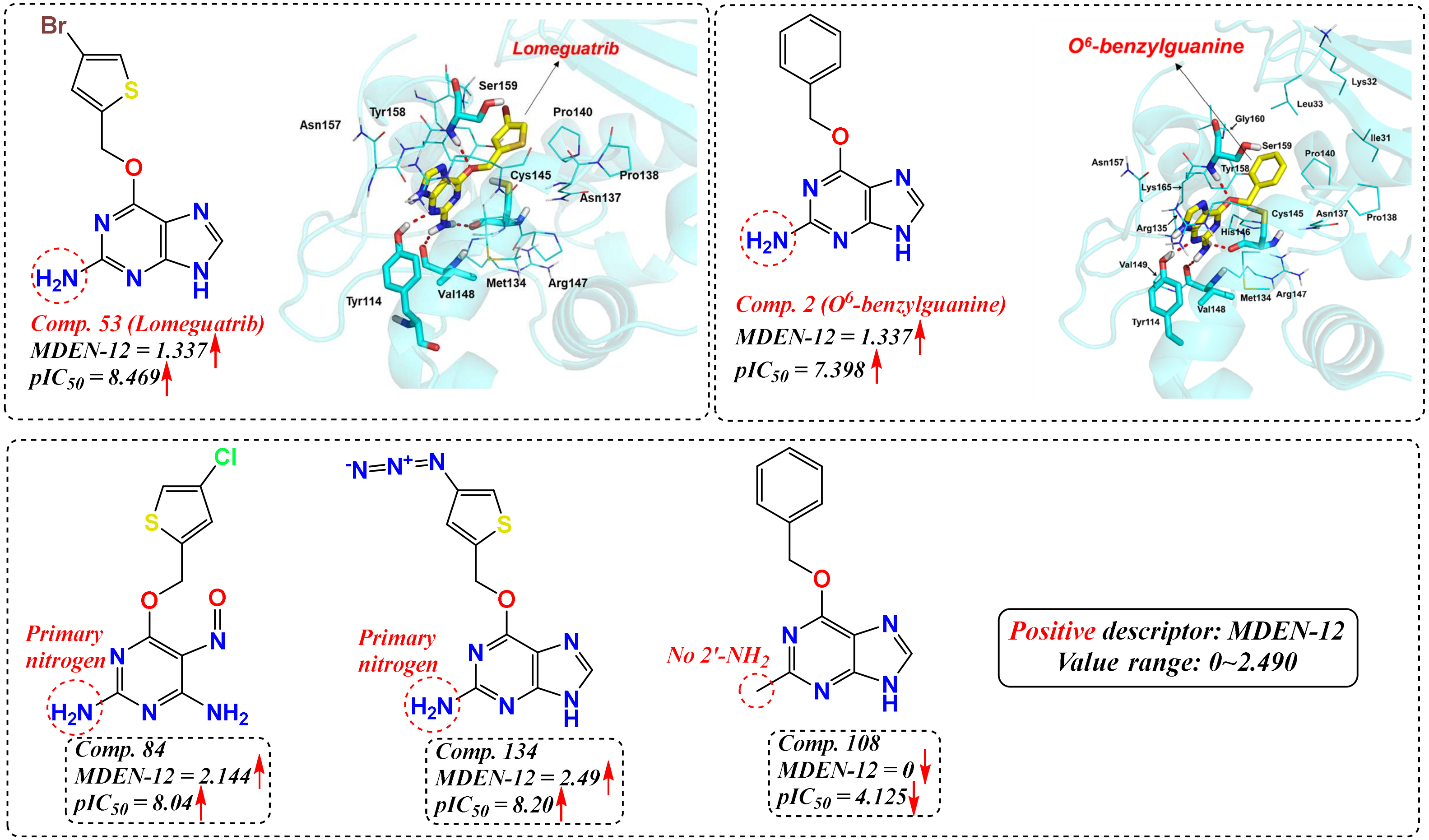

Figure S2. Influence of descriptor MDEN-12 (molecular distance edge between all primary and secondary nitrogens; value range: 0~2.490) on MGMT inactivation potency. Upper panel shows the values for two benchmark inhibitors Lomeguatrib and O^6–benzylguanine and their docked conformations, in which red dashed lines indicate hydrogen bonds. In the figure, red arrow means the descriptor is positively correlated to the MGMT inactivation potency.

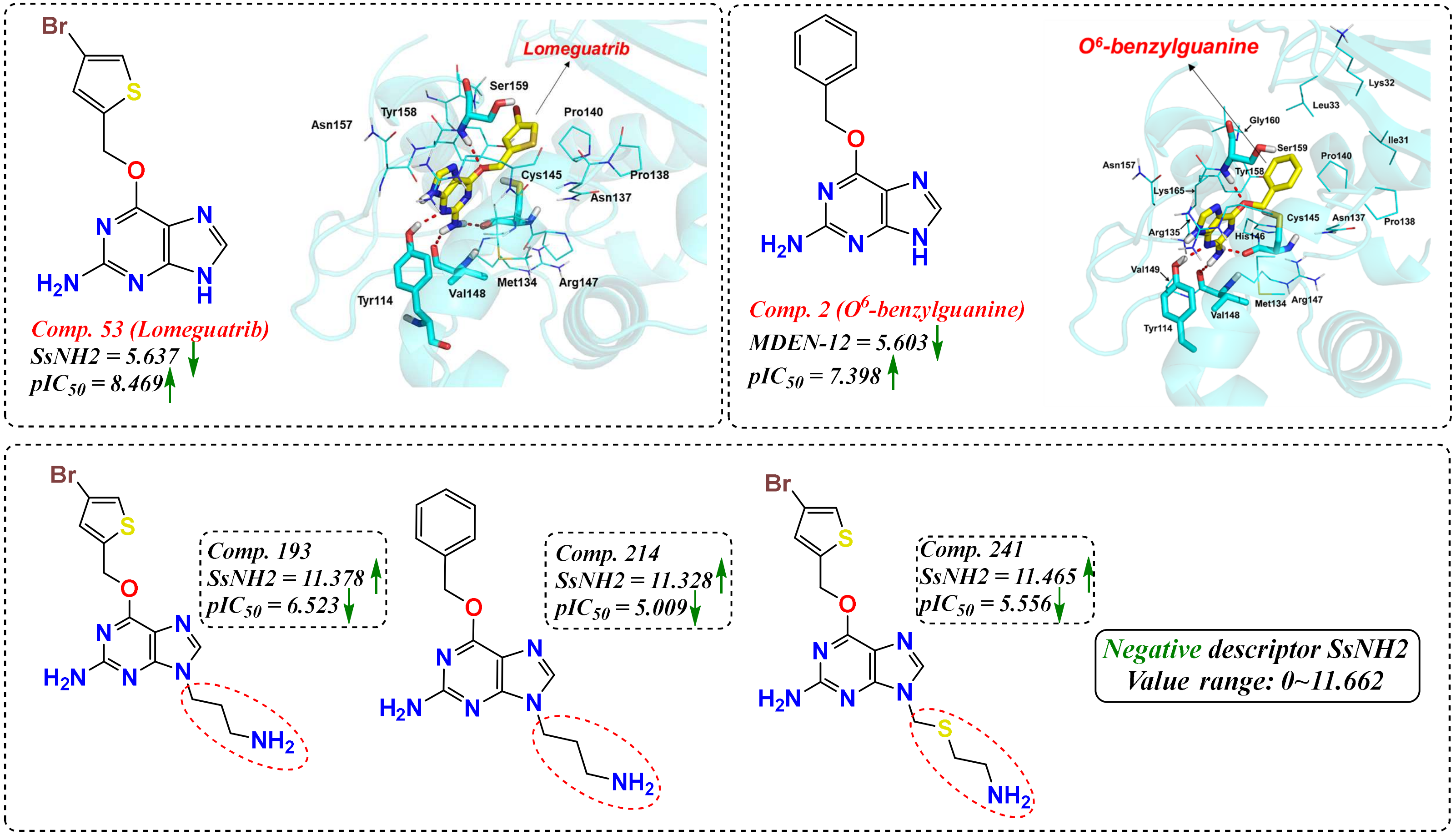

Figure S3. Influence of descriptor SsNH2 (value range: 0~11.662) on MGMT inactivation potency. Upper panel shows the values for two benchmark inhibitors Lomeguatrib and O[6]–benzylguanine and their docked conformations, in which red dashed lines indicate hydrogen bonds. In the figure, green arrow means the descriptor is negatively correlated to the MGMT inactivation potency.

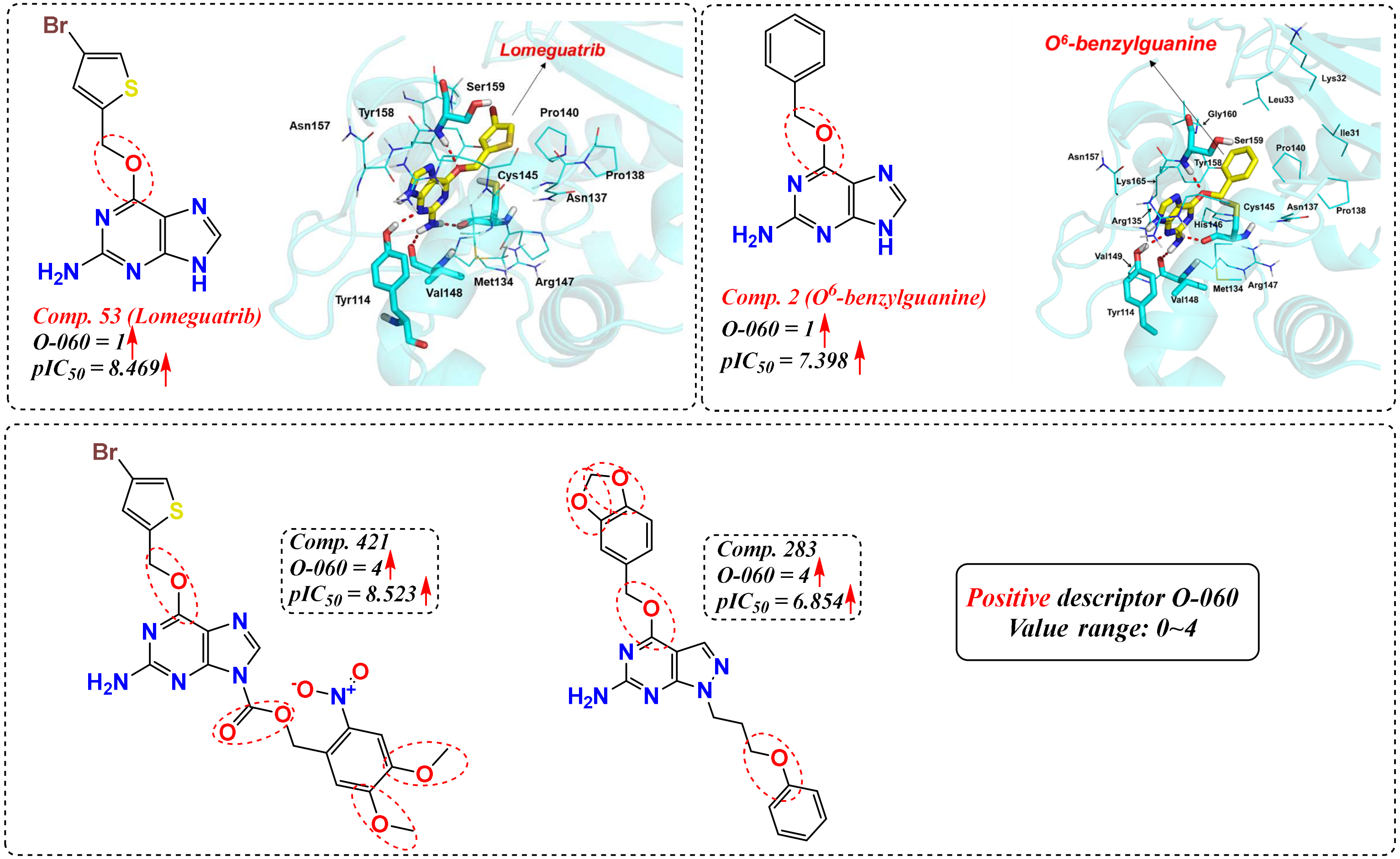

Figure S4. Influence of descriptor O-060 (value range: 0~4) on MGMT inactivation potency. Upper panel shows the values for two benchmark inhibitors Lomeguatrib and O^6–benzylguanine and their docked conformations, in which red dashed lines indicate hydrogen bonds. In the figure, red arrow means the descriptor is positively correlated to the MGMT inactivation potency.

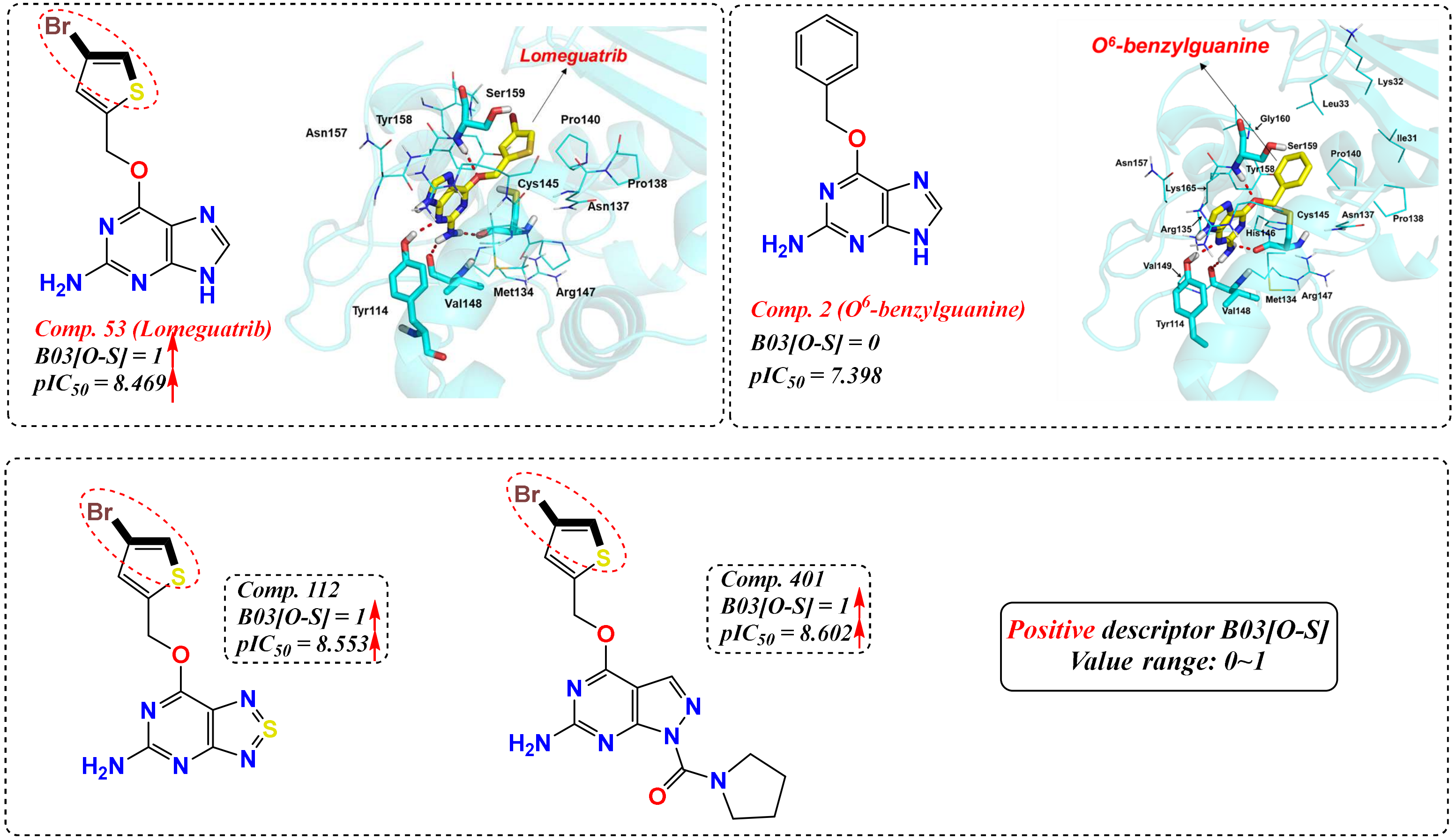

Figure S5. Influence of descriptor B03[O-S] (value range: 0~1) on MGMT inactivation potency. Upper panel shows the values for two benchmark inhibitors Lomeguatrib and O^6–benzylguanine and their docked conformations, in which red dashed lines indicate hydrogen bonds. In the figure, red arrow means the descriptor is positively correlated to the MGMT inactivation potency.

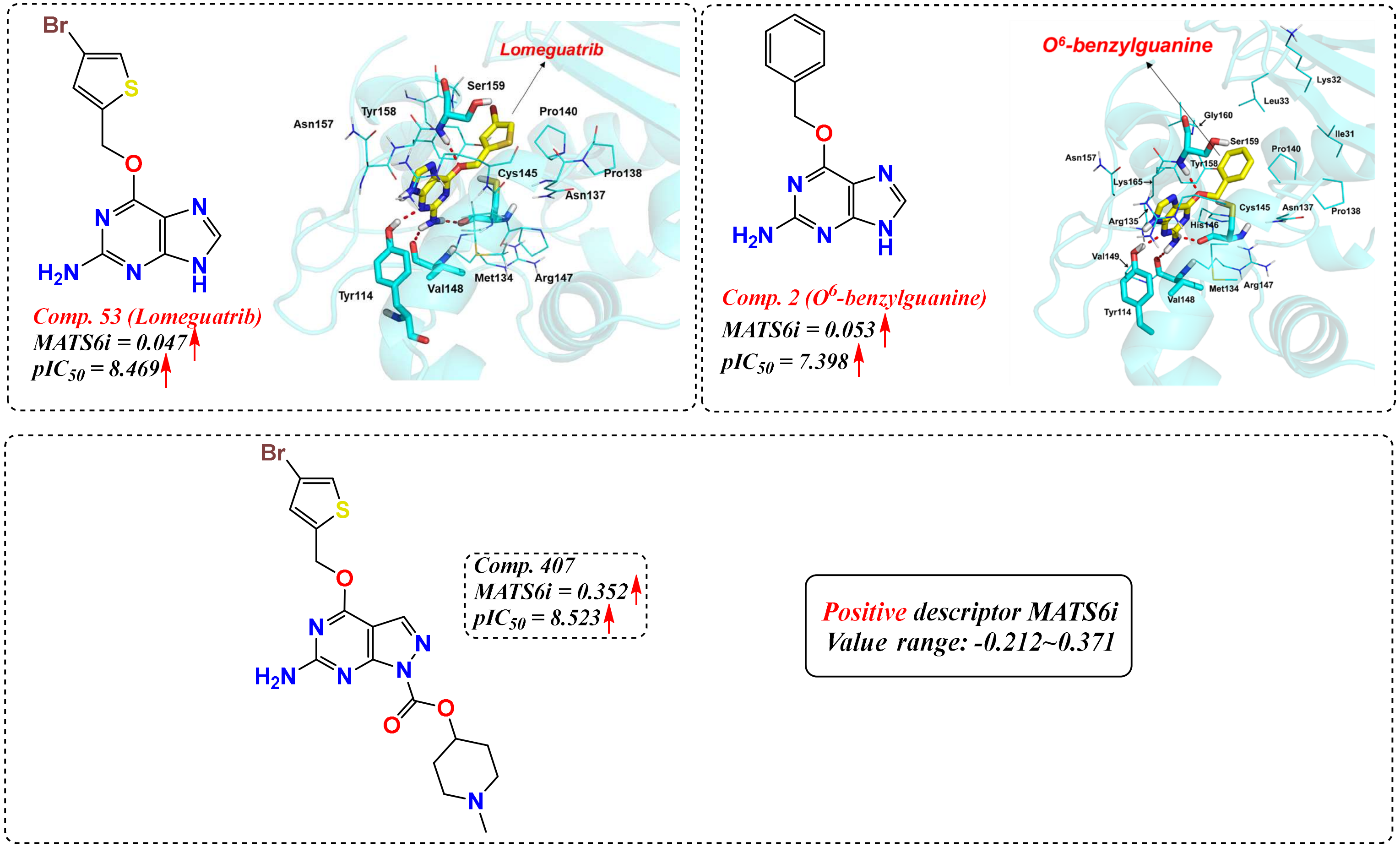

Figure S6. Influence of descriptor MATS6i (value range: -0.212~0.371) on MGMT inactivation potency. Upper panel shows the values for two benchmark inhibitors Lomeguatrib and O^6–benzylguanine and their docked conformations, in which red dashed lines indicate hydrogen bonds. In the figure, red arrow means the descriptor is positively correlated to the MGMT inactivation potency.

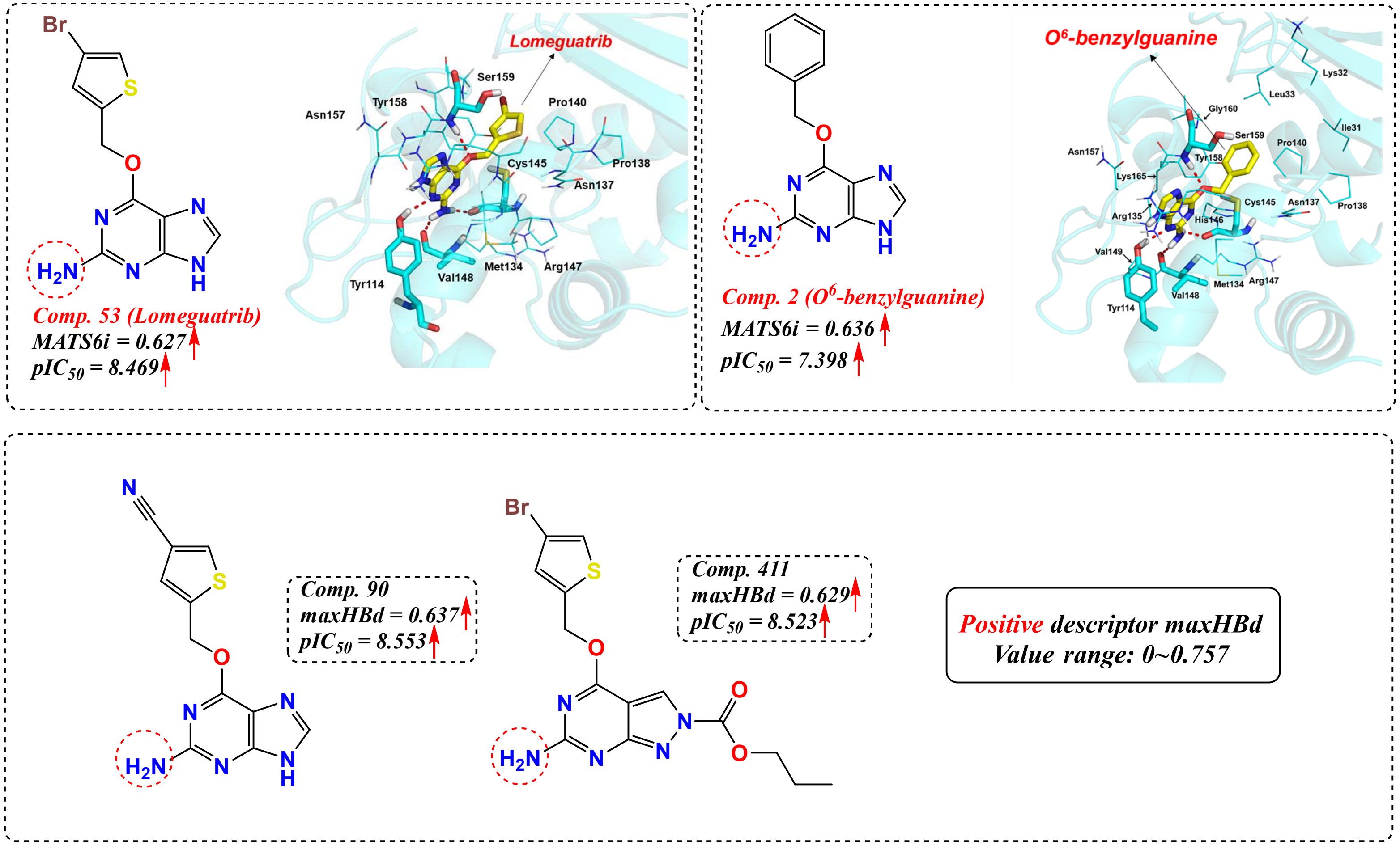

Figure S7. Influence of descriptor maxHBd (value range: 0~0.757) on MGMT inactivation potency. Upper panel shows the values for two benchmark inhibitors Lomeguatrib and O[6]–benzylguanine and their docked conformations, in which red dashed lines indicate hydrogen bonds. In the figure, red arrow means the descriptor is positively correlated to the MGMT inactivation potency.

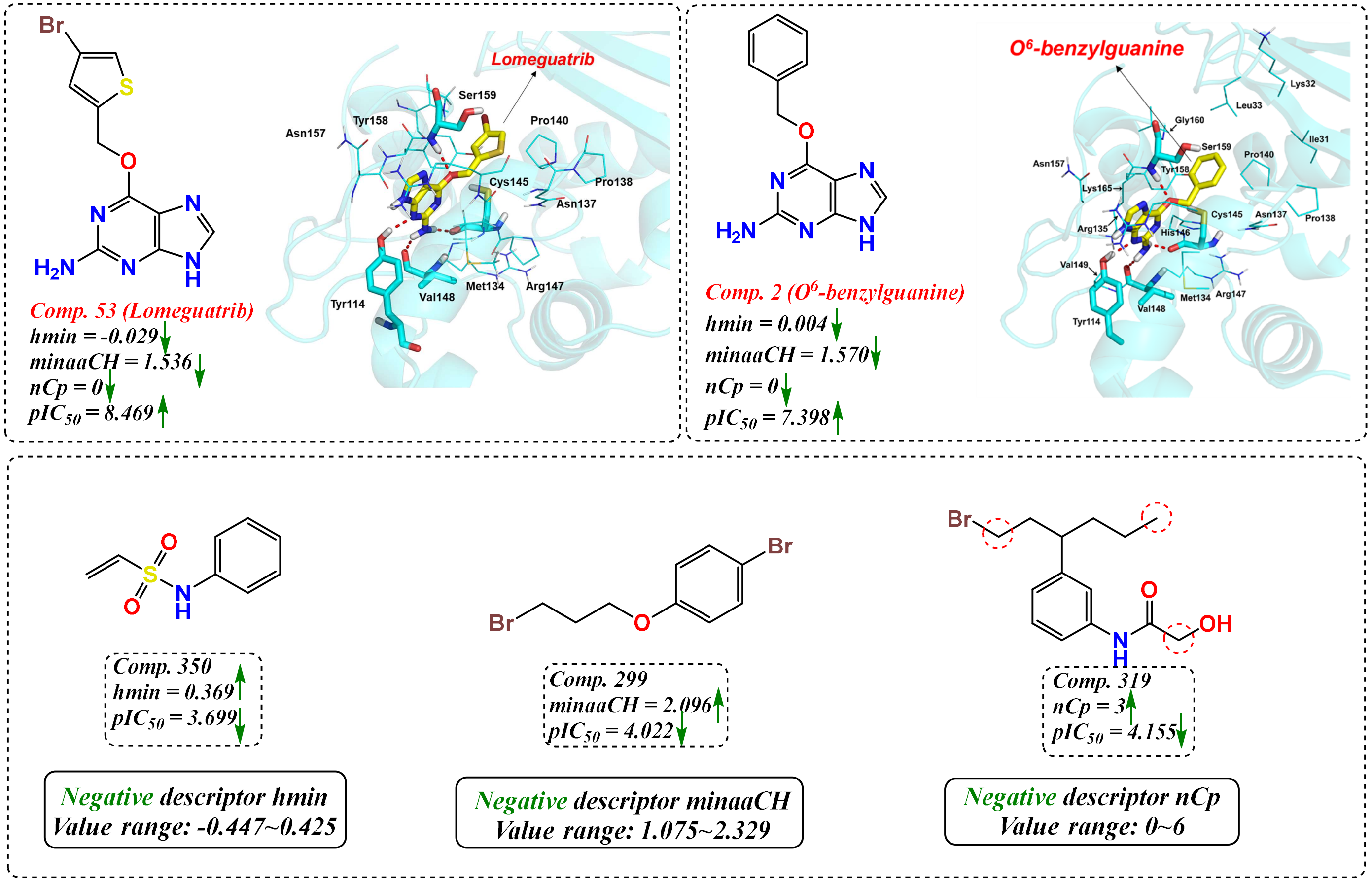

Figure S8. Influence of descriptors hmin (value range: -0.447~0.425), minaaCH (value range: 1.075~2.329) and nCp (value range: 0~6) on MGMT inactivation potency. The three compounds are very different to the majority of compounds (which are base analogs) in the dataset and characterise the three least important descriptors in final model equation. Upper panel shows the values for two benchmark inhibitors Lomeguatrib and O^6–benzylguanine and their docked conformations, in which red dashed lines indicate hydrogen bonds. In the figure, green arrow means the descriptor is negatively correlated to the MGMT inactivation potency.
